# Pleural Effusion Revealing a Diagnosis of Ewing Sarcoma

**DOI:** 10.7759/cureus.20439

**Published:** 2021-12-15

**Authors:** Khaled Ali, Mhd Baraa Habib, Noheir M Taha, Ahmed M Abdalhadi, Riyadh Hammamy

**Affiliations:** 1 Community Medicine, Hamad Medical Corporation, Doha, QAT; 2 Internal Medicine, Hamad Medical Corporation, Doha, QAT; 3 Laboratory Medicine and Pathology, Hamad Medical Corporation, Doha, QAT; 4 Medical Education, Hamad Medical Corporation, Doha, QAT; 5 Medicine, Al Khor Hospital, Al Khor, QAT

**Keywords:** pathology, pulmonology, bone tumors, pleural fluid (pf), ewing sarcoma (es)

## Abstract

Pleural effusion can rarely present as an initial manifestation of Ewing sarcoma. We illustrate a case of a young male adult who was admitted with pleural effusion that led to the diagnosis of Ewing sarcoma.

## Introduction

Ewing sarcoma is a malignant tumor that can arise in bones or soft tissues [1]. Despite its rarity, the Ewing sarcoma family of tumors (EFTs) are the second most common primary bone malignancy among children and adolescents [2]. They mostly affect Caucasians for unknown causes and are extremely uncommon among the African American population [3]. Usually, Ewing sarcoma arises in the long bone of the extremities and the bones of the pelvis. The spine, hands, and feet are affected much less frequently [4]. The most common symptoms are local pain and swelling in the affected area and fever, and pathological fractures may also be seen [5]. We report a case of an unusual presentation of Ewing sarcoma in a young adult who had localized chest pain and pleural effusion.

## Case presentation

A previously healthy 22-year-old Indian male presented to the emergency department with a two-week history of right-sided chest and upper abdominal pain, along with intermittent fever. He denied any other related symptoms. The patient works as a driver and has no family history of malignancy. Physical examination revealed decreased chest sounds on the right lower zone. Diffuse rales and rhonchi were also noted mainly on the right lung field. The remainder of the examination was completely normal.

**Figure 1:**
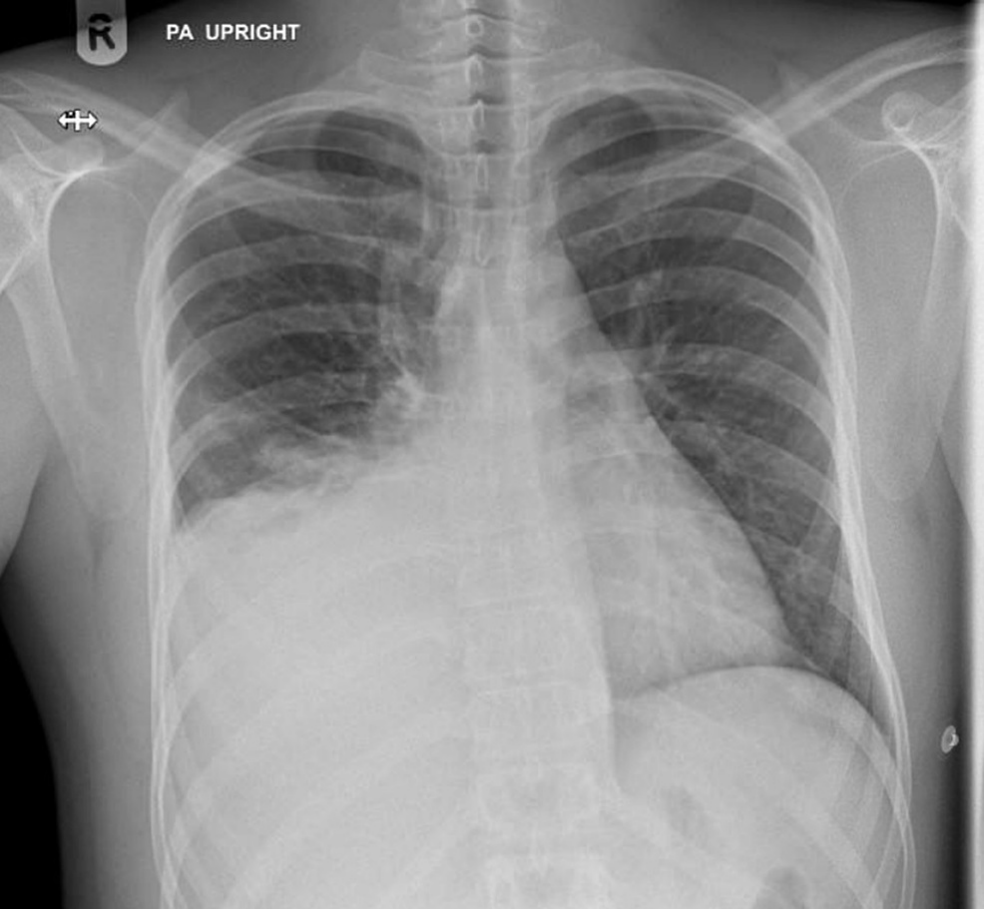
Chest X-ray shows right-sided pleural effusion with the possibility of mass lesion along the right lower hemithorax.

The laboratory tests were within normal limits, except for mild leukocytosis and increased C-reactive protein (Table 1). Chest X-ray revealed right-sided pleural effusion (Figure 1). The patient was admitted as a case of community-acquired pneumonia and started on intravenous antibiotics (amoxicillin/clavulanate, 1000 mg/200 mg). Diagnostic thoracentesis only was done, and it demonstrated hemorrhagic exudative pleural effusion (Table 1). Pleural fluid microscopic examination was negative for malignancy; it showed clusters of reactive mesothelial cells in a background of numerous inflammatory cells, mainly neutrophil polymorph. The abdominal ultrasonography revealed mild to moderate right-sided pleural effusion and a large solid mass along the right lower chest wall with possible downward extension toward the abdominal wall.

**Figure 2:**
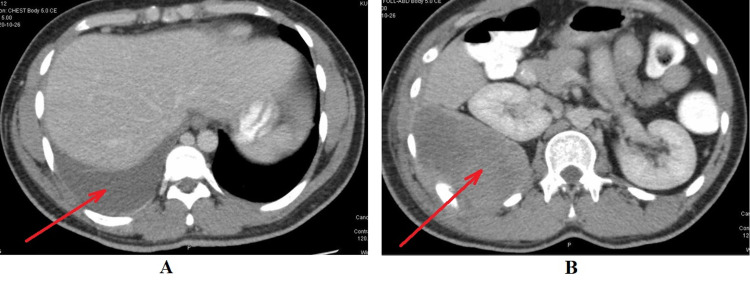
CT scan of the chest, abdomen, and pelvis. A: Mild to moderate right-sided pleural effusion (red arrow). B: Solid heterogeneous soft tissue density lesion (red arrow) along the right lower posterolateral chest wall.

**Figure 3:**
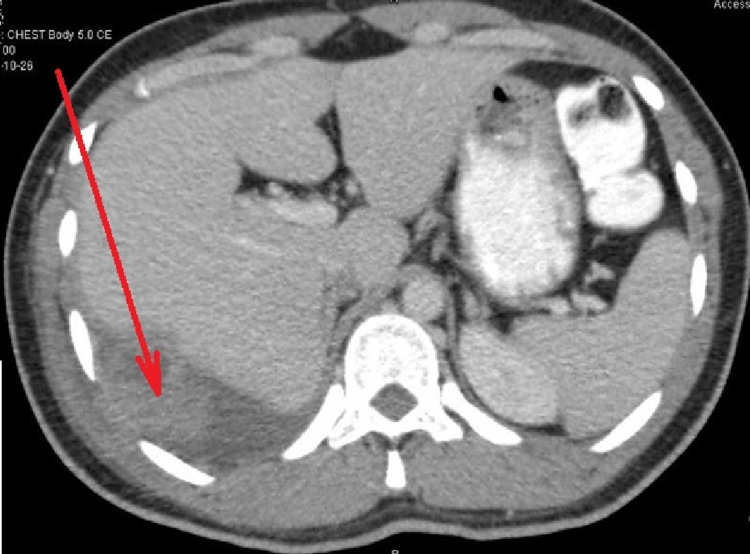
CT scan of the chest, abdomen, and pelvis. It shows that the lesion is seemingly invading the lower posterolateral pleura (red arrow).

**Figure 4:**
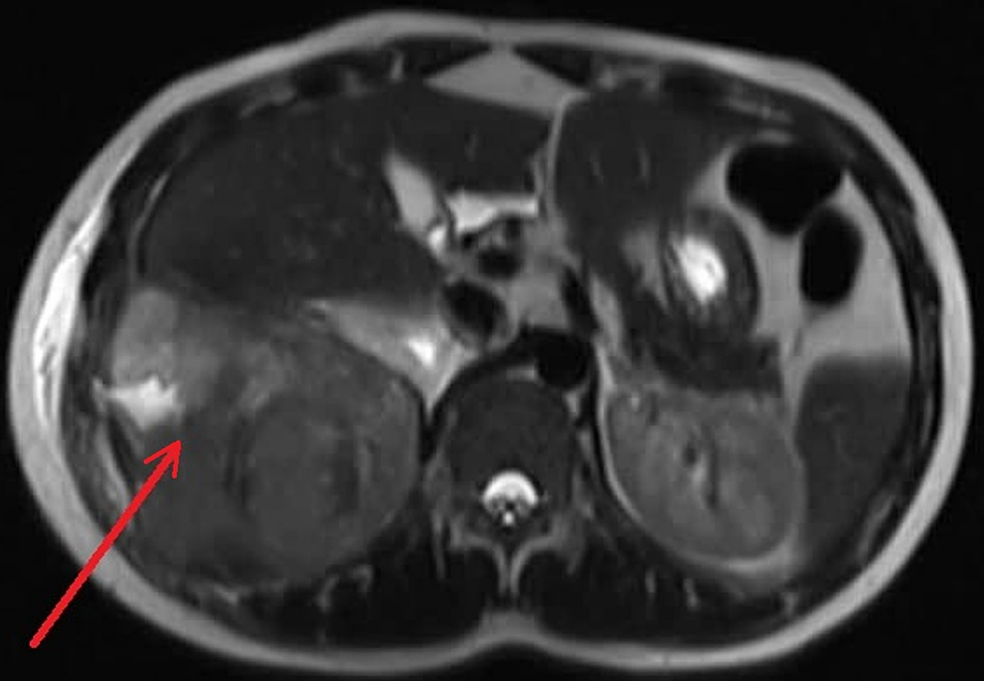
MRI of the abdomen in T2 shows a predominantly solid heterogeneous mass lesion (red arrow) noted in relation to the right lower posterolateral chest wall above and below the diaphragm with hyperintense areas that are suggestive of cystic changes toward the peripheral part.

CT scan revealed an uncalcified solid heterogeneous soft tissue density lesion along the right lower posterolateral chest wall (Figure 2). The soft tissue component is predominantly bulging medially into the lower hemithorax/upper extraperitoneal abdomen. The lesion is seemingly invading the lower posterolateral pleura (Figure 3). MRI of the abdomen and pelvis showed a heterogeneous mass lesion in relation to the right lower posterolateral chest wall, which was suggestive of a soft tissue malignant lesion (Figure 4).

**Table 1:** Laboratory test values.

Detail	Value	Normal range
Blood test results		
White cell count	11.7 × 10^3^/uL	4–10 × 10^3^/uL
Hemoglobin	14.8 g/dL	13–17 g/dL
Creatinine	61 umol/L	62–106 umol/L
NT-proBNP	42 pg/mL	Less than 125 pg/mL
C-reactive protein	182.5 mg/L	0–5 mg/L
Lactate dehydrogenase	Hemolyzed	135–225 U/L
Albumin	34 g/L	35–52 g/L
Glucose random	6.2 mmol/L	3.9–5.5 mmol/L
QuantiFERON-TB Gold Plus	Negative	
Pleural effusion		
Appearance	Bloody	
Red cell count	104,875/uL	
White cell count	8,250/uL	
Neutrophils	51%	
Lymphocytes	38%	
Monocytes	8%	
Glucose	4.5 mmol/L	
Lactate dehydrogenase	148.8 U/L	
pH	7.55	
Protein	47.5 g/L	
Albumin	27.5 g/L	

The suspected mass biopsy results revealed tumoral proliferation formed of undifferentiated small round uniform cells arranged into irregular sheets separated by fibrous bands. The tumor cells have scant clear cytoplasm with indistinct cell borders and hyperchromatic nuclei with inapparent nucleoli. The pathology findings indicated a malignant small round cell tumor, which is compatible with Ewing sarcoma (Figures 3, 4). The plan was to start chemotherapy, but the patient traveled back to have the treatment in his home country.

**Figure 5:**
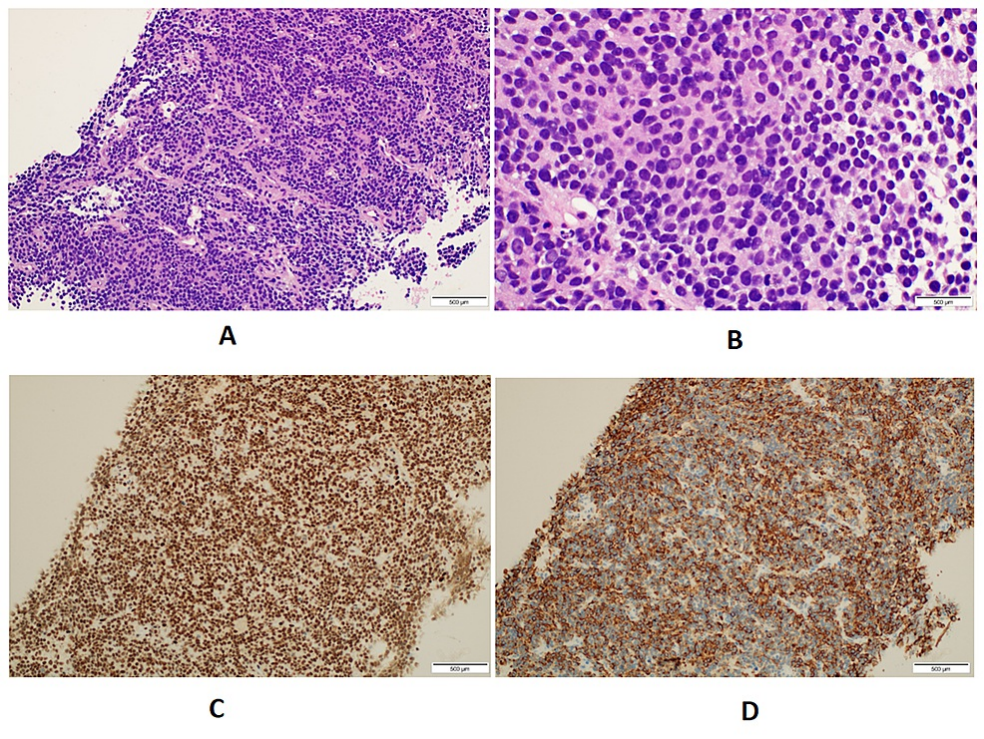
Pathological findings of the mass biopsy: A: Sheets of the densely cellular undifferentiated tumor with stromal fibrous strands (H&E: ×20). B: Sheets of small, round, uniform cells with scant clear cytoplasm and indistinct cell membranes with occasional mitosis. C: Immunohistochemical stain for FLI-1 shows diffuse and strong nuclear staining of the tumor cells (H&E: ×20). D: Immunohistochemical stain for CKAE1/3 shows diffuse and strong cytoplasmic staining of the tumor cells (H&E: ×20).

**Figure 6:**
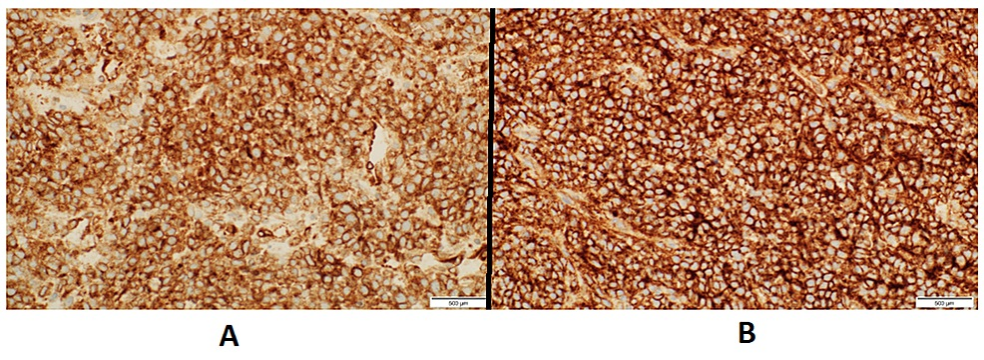
Pathological findings of the mass biopsy: A: Immunohistochemical stain for BCL-2 shows diffuse and strong membranous staining of the tumor cells (H&E: ×40). B: Immunohistochemical stain for CD99 shows diffuse and strong membranous staining of the tumor cells (H&E: ×40).

## Discussion

Extraskeletal Ewing sarcoma is well known among adolescents, which usually presents with localized mass [6]. Pleural effusion is a quite rare presentation of Ewing sarcoma and can be easily missed especially in patients who are from endemic areas of tuberculosis, which is usually one of the main causes of exudative pleural effusion [7]. This advocates the importance of considering Ewing sarcoma in the cases of bloody pleural effusion in young adult patients. Histopathological examination of any hemorrhagic pleural fluid is crucially important in such cases to avoid the catastrophic overlook of malignancy [8]. In our case, although the pleural fluid was negative for malignant cells, it was eventually found to have resulted from the malignant mass of Ewing sarcoma.

The definition of malignant pleural effusion clearly states that the fluid must contain malignant cells [9]. However, in the presence of a highly suspected mass, a pleural fluid with negative malignant cells is called a para-malignant effusion [10]. Para-malignant pleural effusion can range from concordant exudate to transudate with mediastinal lymphadenopathy as the most common cause [11]. It occurs as a result of multiple mechanisms, including when malignant cells lose adhesion and dislodgement from the primary tumor site, blood vessel wall penetrating/migrating through the pleura, and induction of angiogenesis [12,13]. In our case, Ewing sarcoma was located in the chest wall and caused a para-malignant pleural effusion, which is a very rare behavior and also a rare location to arise from.

Other extremely rare locations of Ewing sarcoma were reported, such as the parotid gland [14], jejunum [15], and stomach [16]. Moreover, Ewing sarcoma was reported to be localized intracranially in a four-year-old boy [17]. Another case of epidural Ewing sarcoma in a 45-year-old woman that caused paresthesia and urinary retention was also reported as one of the rarest presentations of Ewing sarcoma [18].

Although both skeletal and extraskeletal Ewing sarcoma have generally the same treatment approaches, extraskeletal Ewing sarcoma tends to be more aggressive with a higher probability for local recurrence and metastasis [19]. Interestingly, however, overall survival of five years was found to be the same in both types [20]. Our patient refused to commence the chemotherapy regimen and preferred to fly back to his home country; therefore, we could not follow him up and reevaluate his case.

## Conclusions

We demonstrate in this case the wide variety of Ewing sarcoma presentations, with more focus on pleural effusion as an interesting and unusual feature, especially in young adult patients. These cases can be overlooked, especially when the fluid is negative for malignant cells, which may delay the diagnosis and result in an awfully bad prognosis.
